# Supplementary material for: Improving course evaluation processes in higher education institutions: a modular system approach
Source: PeerJ Comput Sci. 2025 Aug 28;11:e3110. doi: 10.7717/peerj-cs.3110 (PMC12453702; doi:10.7717/peerj-cs.3110)
Supplement: Supplemental Information 1 [file peerj-cs-11-3110-s001.docx]

**Course Evaluation Report**

**General Information**

Term: 2023/2024 Spring Semester

Course Code/Name: ………………………

Course Instructor: ………………………

Note: There are 430 course evaluation surveys for the course. Since 37% of these surveys consist of inconsistent data, this report is composed of the remaining 270 course evaluation surveys.

**Course Analysis**

**Emotional State**

81% of the comments made for the course are positive, 11% are negative, and 8% are neutral.

•Positive

•Negative

•Neutral

**Survey Analysis**

The average result of the course survey is 3.49. The highest survey score is 3.53 for Question 1, while the lowest survey score is 3.44 for Question 5.

**Question 1:** The instructor's explanation regarding how the course will be conducted.
**Question 5:** The clarity of the materials used in the course.

**Examples of Positive Comments:**

- The content of the course was consistent with the materials used and the instructor's explanations.
- I love the instructor very much; they were very attentive during the lessons. My exam results weren’t as good as I hoped, but I hope I don’t end up with a very low grade.
- I was very satisfied with the course delivery and the skills gained from both the midterm and final exams. I sincerely thank our instructor.

**Examples of Negative Comments:**

- We were supposed to have 100-minute sessions instead of 50 minutes, but we usually finished in a total of 70 minutes, causing many students to rush through experiments.
- Instructor, you reply to emails late. You delay certain things, and this impacts our study schedule—for example, uploading the presentations late. Additionally, I find your teaching method unsuitable. Sorry, but you often just read from the book during class. At times, you literally just sat and read the book. Initially, I was very excited about this course, but I lost that excitement after the second session. I think the course should be delivered with a more engaging tone, fun approach, and an interesting strategy.
- Unfortunately, I feel that we were left on our own with the topics we were supposed to learn, and we were constantly compared to the other section. We faced remarks like, "You'll fail the course anyway," which weakened our interest in the course and lowered our motivation. Seeing that I couldn’t perform as well in this course as I did in my other courses upset me, and my GPA dropped just because of this. I hope these issues won’t occur again next semester.

**Examples of Neutral Comments:**

- In-person lessons are not efficient; there should be more practical applications.
- More practical activities could be incorporated into the lessons.
- None.
