# Supplementary material for: Improving course evaluation processes in higher education institutions: a modular system approach
Source: PeerJ Comput Sci. 2025 Aug 28;11:e3110. doi: 10.7717/peerj-cs.3110 (PMC12453702; doi:10.7717/peerj-cs.3110)
Supplement: Supplemental Information 4 [file peerj-cs-11-3110-s004.docx]

| Original (Turkish) Version |
| --- |
| Dersin nasıl yürütüleceğine yönelik ders sorumlusunun bilgilendirmesi.  Dersin kazanımlarına yönelik ders sorumlusunun paylaşımı.  Ders izlencesinde ele alınan konuların kazanımlarla uyumu.  Derste kullanılan materyallerin (ders notları, sunum slaytları, kitap, videolar, görsel ögeler vs.) ders içeriği ile uyumu.  Derste kullanılan materyallerin (ders notları, sunum slaytları, kitap, video, görsel ögeler vs.) anlaşılırlığı.  Öğretim elemanının derste kullandığı dilin anlaşılırlığı.  Öğretim elemanının öğrenmelerimize yönelik geri bildirimlerinin öğrenmeye katkısı.  Öğretim elemanının derse hazır oluş düzeyi.  Öğretim elemanının konuya hakimiyeti.  Öğretim elemanının derse ayrılan tüm zamanı etkili kullanması.  Öğretim elemanının derse katılan öğrencilerle olan iletişimi.  Öğretim elemanına ders zamanı dışında farklı platformlarda ulaşılabilirlik düzeyi.  Ödevlerin ve dönem boyunca yapılan değerlendirmelerin öğrenmeye olan katkısı.  Ödevlerin, sınavların, projelerin dersin içeriği ile uyumu.  Öğretim elemanının öğrencileri derse katılmaya teşvik etmesi.  Öğretim elemanının bireysel öğrenme farklılıklarını dikkate alma düzeyi. |
| English Version |
| Informing students about how the course will be conducted by the course instructor.  Sharing the course outcomes by the course instructor.  Alignment of the topics covered in the course syllabus with the learning outcomes.  Compatibility of the materials used in the course (lecture notes, presentation slides, books, videos, visual elements, etc.) with the course content.  Clarity of the materials used in the course (lecture notes, presentation slides, books, videos, visual elements, etc.).  Clarity of the language used by the instructor during the course.  The contribution of the instructor's feedback to learning.  The instructor's level of preparedness for the course.  The instructor's expertise in the subject matter.  Effective use of the allotted course time by the instructor.  Communication between the instructor and the students attending the course.  Accessibility of the instructor outside class hours through different platforms.  Contribution of assignments and assessments throughout the semester to learning.  Alignment of assignments, exams, and projects with the course content.  The instructor's efforts to encourage student participation in the course.  The instructor's consideration of individual learning differences. |
|  |
